# Diagnosis of Familial Wolf-Hirschhorn Syndrome due to a Paternal Cryptic Chromosomal Rearrangement by Conventional and Molecular Cytogenetic Techniques

**DOI:** 10.1155/2013/209204

**Published:** 2013-02-03

**Authors:** Carlos A. Venegas-Vega, Fernando Fernández-Ramírez, Luis M. Zepeda, Karem Nieto-Martínez, Laura Gómez-Laguna, Luz M. Garduño-Zarazúa, Jaime Berumen, Susana Kofman, Alicia Cervantes

**Affiliations:** ^1^Servicio de Genética, Hospital General de México, Dr. Balmis No. 148, Colonia Doctores, 06726 México, DF, Mexico; ^2^Facultad de Medicina, Universidad Nacional Autónoma de México, México, DF, Mexico; ^3^Departamento de Medicina Genómica, Hospital General de México, Dr. Balmis No. 148, Colonia, Doctores, 06726 México, DF, Mexico

## Abstract

The use of conventional cytogenetic techniques in combination with fluorescent *in situ* hybridization (FISH) and single-nucleotide polymorphism (SNP) microarrays is necessary for the identification of cryptic rearrangements in the diagnosis of chromosomal syndromes. We report two siblings, a boy of 9 years and 9 months of age and his 7-years- and 5-month-old sister, with the classic Wolf-Hirschhorn syndrome (WHS) phenotype. Using high-resolution GTG- and NOR-banding karyotypes, as well as FISH analysis, we characterized a pure 4p deletion in both sibs and a balanced rearrangement in their father, consisting in an insertion of 4p material within a nucleolar organizing region of chromosome 15. Copy number variant (CNV) analysis using SNP arrays showed that both siblings have a similar size of 4p deletion (*~*6.5 Mb). Our results strongly support the need for conventional cytogenetic and FISH analysis, as well as high-density microarray mapping for the optimal characterization of the genetic imbalance in patients with WHS; parents must always be studied for recognizing cryptic balanced chromosomal rearrangements for an adequate genetic counseling.

## 1. Introduction

Current diagnosis of chromosomal syndromes should include a combination of conventional cytogenetic techniques with molecular cytogenetic methods, particularly fluorescent *in situ* hybridization (FISH), as well as modern genomic applications such as copy number variations (CNVs) analysis by single-nucleotide polymorphism (SNP) or comparative genomic hybridization (aCGH) microarray techniques. The laboratory methods employed to achieve an adequate diagnosis of a familial case of Wolf-Hirschhorn syndrome (WHS, OMIM194190) exemplifies how the conventional, molecular and genomic techniques are complementary and useful to provide an appropriate genetic counseling in chromosomal syndromes.

 Wolf-Hirschhorn syndrome affects at least 1/50,000 newborns and presents a broad range of clinical manifestations. WHS is characterized by a typical craniofacial appearance, growth delay, mental retardation, hypotonia, and seizures [1]. In the majority of cases (50–60%), WHS is caused by “pure” *de novo* terminal or interstitial deletions in 4p16; unbalanced translocations (45%), either *de novo* or inherited from a balanced rearrangement (~15%), and other complex cytogenetic findings (>1%) such as a chromosome 4 ring, del(4p) mosaicism, or a duplication/deletion rearrangement derived from a chromosome 4 inversion [2, 3] have been observed as well. In a high proportion of the WHS patients (25–30%), the chromosomal abnormality is cryptic and not detectable by conventional cytogenetic techniques. In cases of clinical suspicion of WHS in a patient with normal karyotype, additional FISH studies of the sub-telomeres and the WHS critical region (WHSCR) must be performed [4]. 

 The high degree of variation in the clinical presentation of WHS has been attributed to differences in the size of the 4p deletion, the presence of a partial trisomy from the segregation of a chromosomal translocation or inversion, allelic differences or multifactorial inheritance [2–5]. The majority of familial cases have been associated with parental chromosomal balanced translocations, particularly t(4p; 8p), which represents a distinct genetic entity [2, 5, 6]. Chromosomes 7p, 11p, 12p, and Dp/Gp, have also frequently been implicated in 4p inherited or *de novo* rearrangements [2, 5, 7–10]. We describe two sibs with a classic WHS phenotype and 4p16.1-p16.3 deletions (~6.5 Mb) due to the segregation of a paternal balanced rearrangement, characterized by karyotyping, FISH, and microarray copy-number analysis.

## 2. Materials and Methods

### 2.1. Clinical Report

**Figure 1:**
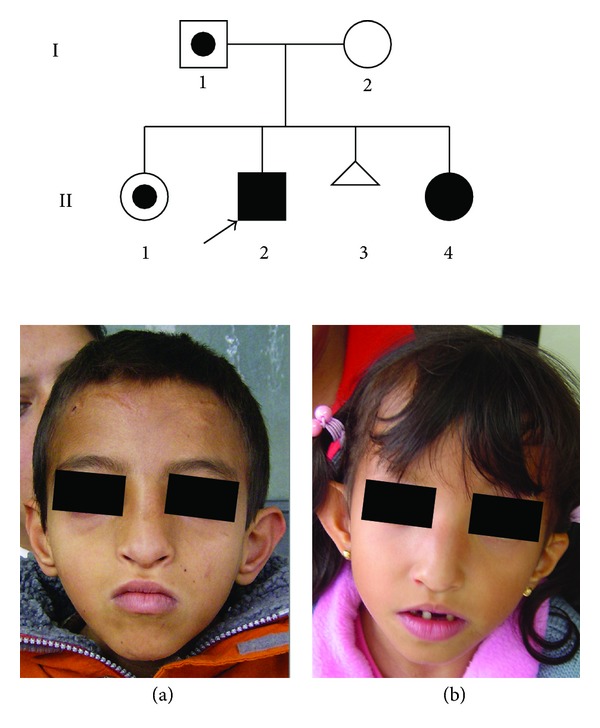
Family pedigree and patient profiles: (a) II.2 at the age of 9 years 9 months; (b) II.4 at the age of 7 years 5 months. Both patients exhibited typical WHS phenotypes.

The family pedigree is shown in Figure 1. The parents were a healthy, young, non-consanguineous couple. II:1 is a healthy 11-year old girl. The propositus (II:2) is a 9 years and 9 months of age boy, born at 37.5 gestation weeks by cesarean section due to fetal distress; birth weight: 2,125 g, height 43 cm (both <3rd centile). Hypotonia was noted at birth. At 7 months of age, the patient developed generalized tonic-clonic seizures. Clinical examination at 9 years and 9 months of age revealed psychomotor retardation; height 124 cm, weight 15 kg, and OFC 45.3 (all <3rd centile). He displayed facial features typical of WHS (Figure 1(a)). Psychological examination by WISC-R revealed a global IQ of 25. II:4 is a 7-years and 5-month old girl, born at 37.2 weeks by cesarean section, birth weight 2,100 g and height 42 cm (both <3rd centile). She showed clinical findings similar to those of her brother (Figure 1(b)), and renal ultrasound reported left kidney malrotation. Her global IQ was 30. The clinical features of both patients are described in Table 1. Initial conventional cytogenetic analysis by GTG banding (400–700 bands) revealed a 4p16 deletion in both sibs, suggesting a parental chromosomal balanced rearrangement.

### 2.2. Cytogenetic and FISH Analysis

 Chromosome analyses on lymphocytes by GTG (400–700 bands) and NOR banding were performed according to standard protocols. FISH was performed using LSI WHSCR1 Spectrum Orange and CEP 4 Spectrum Green probes and ToTelVysion Mixtures number 4 (4p Spectrum Green, 4q Spectrum Orange, 21q Spectrum Green/Orange, and LSI AML1 Spectrum Aqua) and number 10 (10p Spectrum Green, 10q Spectrum Red, 15q Spectrum Green/Orange and LSI PML Spectrum Aqua) from Vysis Abbot, Inc. (Abbot Park, IL, USA), according to the procedures described by the manufacturer.

### 2.3. Microarray Analysis

High purity genomic DNA was extracted from 3 mL whole blood using the Versagene DNA Purification kit (Gentra Systems Inc., Minneapolis, MN, USA). Genomic mapping was performed on the affected sibs and parents using the Genome-wide human SNP array 5.0 set (Affymetrix Inc., Santa Clara, CA, USA), according to the protocol supplied by the manufacturer. Genotyping Console 4.1 (Affymetrix Inc.) was used for quality assessment and genotyping of the data. The QC call rate by the BRLMM-P algorithm was over 93%. CNV analysis was performed using SNP & Variation Suite 7.5.6 software (Golden Helix Inc., Bozeman, MT, USA). Patients' data were normalized against a reference set generated in our laboratory, consisting of 71 healthy subjects including the patients' parents. The copy number analysis method (CNAM) was used to identify the CNV segments with a moving window of 10,000 markers in a univariate basis. Mapping was carried out based on the human genome assembly Feb 2009 (GRCh 37/hg19) (NCBI Reference Sequence (RefSeq) http://www.ncbi.nlm.nih.gov/RefSeq/).

## 3. Results

### 3.1. Cytogenetic and FISH Analysis

**Figure 2:**
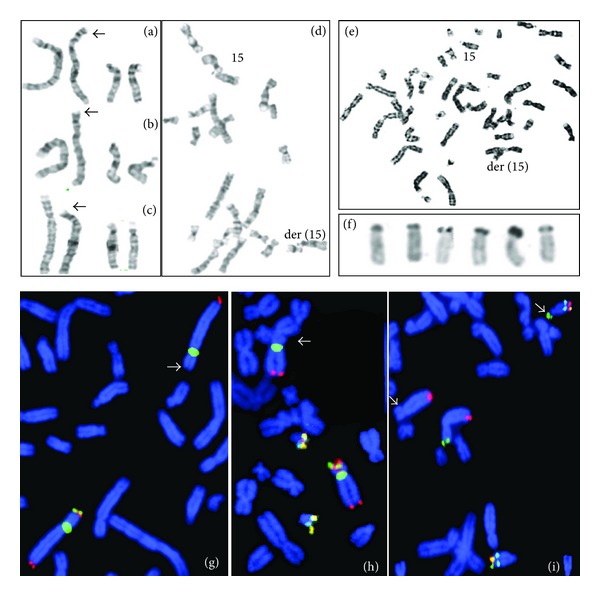
Partial karyotypes from the family. (a) II:2, (b) II:4, and (c) I:1: chromosomes 4 and 15 with GTG banding. (d) and (e) Partial metaphases from the father showing chromosome 15 and der(15) associated with acrocentric chromosomes. (f) Group D metaphase chromosomes from the father demonstrating active Ag-NOR in all chromosomes, including der(15). (g) II:2 and (h) II:4 FISH with LSI WHSCR1 (orange), subtelomeric 4p (green) probes and controls CEP 4 (green), 4q subtelomeric (orange), 21q (orange/green) and LSI AML1 (aqua), showing the absence of both 4p signals on one chromosome 4. (i) I:2 (father) FISH with ToTelVysion mixtures 4 and 10, showing a green 4p subtelomeric signal on 15p.

High-resolution GTG banding on the affected children revealed a 4p16.1 deletion (Figures 2(a) and 2(b)). FISH using WHSCR1 and 4p subtelomeric probes confirmed the loss of both sequences (Figures 2(g) and 2(h)). The mother's karyotype was normal, while the phenotypically normal father carries a derivative chromosome 4 and an apparent heteromorphism in both chromosomes 15 (Figure 2(c)). FISH using ToTelVysion Mixtures 4 and 10 showed that 4p subtelomeric signal was located on the short arm of one chromosome 15 (Figure 2(i)). Ag-NOR banding was negative on 4p, and no association of der(4) with acrocentric chromosomes was observed; der(15) was positive for Ag-NOR and acrocentric association (Figures 2(d), 2(e) and 2(f)) confirming an insertion from 4p to 15p. The other chromosome 15 showed an increased stalk on its short arms (Figures 2(e) and 2(f)). Both affected children inherited this chromosome 15pstk+ (Figures 2(a) and 2(b)). The father's final karyotype was 46,XY,ins(15;4)(p12;p16.1p16.3).ish ins(15; 4) (D4S3359+,PML+,D15S936+; D4S3359-,D4S2930+). II: 1 inherited the same balanced rearrangement from her father (data not shown).

### 3.2. Microarray Analysis

**Figure 3:**
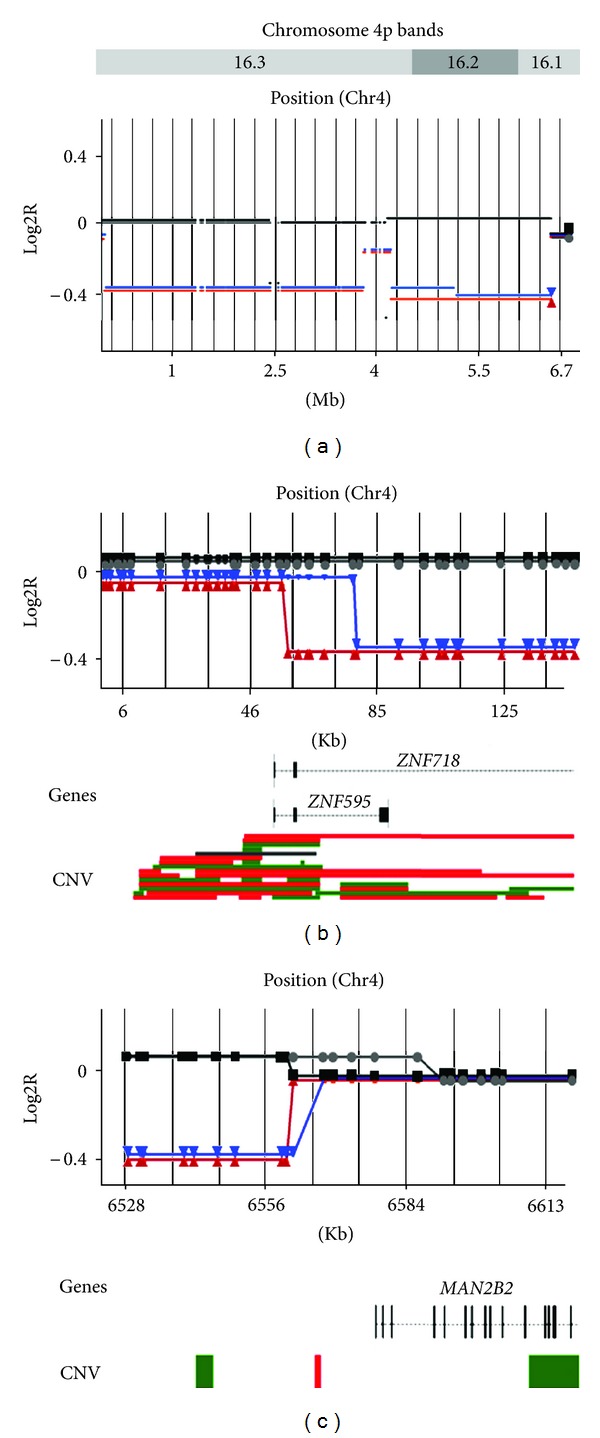
(a) Deletion involving chromosome bands 4p16.1-p16.3 was confirmed by microarray mapping of the propositus (*▼*), his younger sister (▲) and both parents (father (▪) and mother (●)). (b) The affected patients display differential telomeric break points, which occur at a variable region including genes *ZNF718* and* ZNF595*. (c) The centromeric break points in both patients were located >15 kb upstream of the *MAN2B2* transcriptional start site (pos. 6576902). Gene (RefSeq) and CNV (DGV) annotation maps are shown below. CNV gain regions are indicated in red, losses in green, and gain/losses in gray. Log2R, logarithmic value of the sample to reference ratio.

CNV analysis confirmed a similar 4p deletion in both siblings, 6.48 Mb in the propositus, and 6.50 Mb in his affected sister. The minimal deletion positions were from nt.69,535 to 6,546,304 and from nt.58,388 to 6,560,313, respectively (Figure 3(a)). These include WHSCR and WHSCR2. The telomeric break points affected the *ZNF718* and *ZNF595* genes in both sibs; however an 11.1 Kb difference was observed between these (Figure 3(b)). The distal region of 4p is highly variable (database of Genomic Variants http://projects.tcag.ca/variation/), and the children inherited a different chromosome 4 from their mother, as documented by the SNP genotyping analysis (data not shown). The centromeric break point differs by 14 Kb between sibs, and in both cases the gene *MAN2B2* maps outside the deletion, at least 15.2 Kb from its start point (Figure 3(c)). Acrocentric p arms are not represented in the 5.0 SNP array. 

## 4. Discussion

Three clinical categories of WHS have been defined according to the size of the 4p deletion: (1) <3.5 Mb, linked to a mild form, (2) between 5 and 18 Mb, associated with the classical phenotype observed in our patients, and (3) >22 Mb, causing a severe form [5]. The pathogenesis of WHS is multigenic, and genotype-phenotype correlation studies may clarify the role of specific genes on 4p in the disease etiology [2, 4]. CNV analysis in our patients revealed a similar 4p deletion of ~6.5 Mb, with the common deleted segment spanning from 69,535 Kb to 6,546,304 Mb, that is, 4p16.1 to 4p16.3 (Figure 3(a)). The deletion affects at least 70 genes, including the 200 kb critical region for the typical WHS phenotype [11], and the candidate genes *LETM1, FGFRL1* and *WHSC1*, which have been associated with seizures, some facial findings, distinctive facial features and growth delay, respectively [4, 12]. The genes *ATP5I, FGFR3, HTT, MSX1* and *PPP2R2C* are likely haploinsufficient (Decipher database http://decipher.sanger.ac.uk), and could also be relevant to the WHS phenotype. 

**Figure 4:**
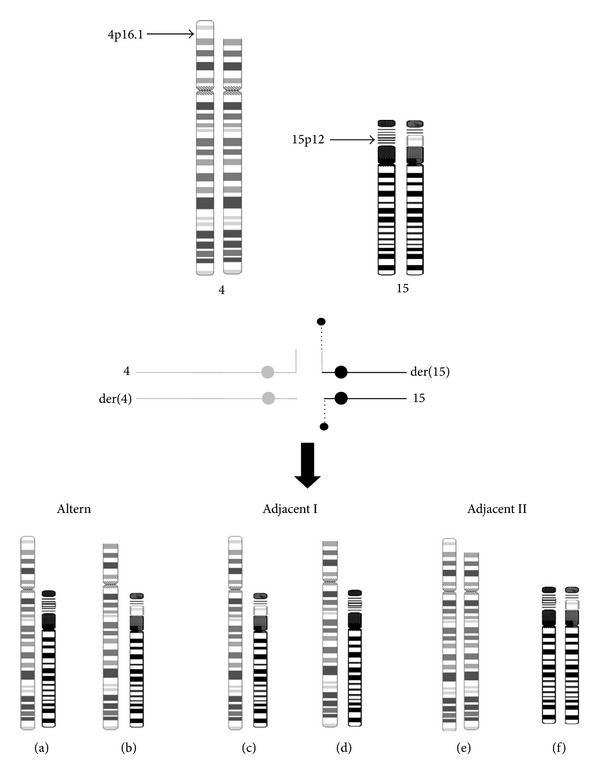
Chromosomes 4 and 15 ideograms showing the paternal insertion and its meiotic segregation. The affected siblings were the product of an adjacent I segregation.

 Different types of chromosomal rearrangements are associated with WHS; among these, inherited unbalanced translocations are frequently maternal, while *de novo* unbalanced translocations are usually paternal, with the exception of the t(4;8) [2, 5, 13]. In our patients, we identified an isolated 4p deletion due to a paternal balanced insertion (Figure 4). To our knowledge, this rearrangement has not been previously reported in WHS. 

 Recently, it has been suggested that chromosomal insertions are more frequent (1 : 500) [14–16] than previously reported (1 : 80000) [17]. These rearrangements involve three chromosome breakage events that can be intra- or interchromosomal. The use of FISH to confirm deletions and/or duplications detected by microarrays showed that these genomic imbalances resulted from the segregation of a parentally balanced insertion [14, 15]. Interestingly, the short arms of acrocentric chromosomes are frequently involved in these rearrangements, especially the NOR of chromosomes 15 and 22, and they are often cryptic when present in an unbalanced form [14]. Genomic studies have demonstrated that different gene families and certain satellite repeats, like the olfactory receptor gene family and the terminal 4p repeats, constitute nucleolus-associated chromatin domains that interact with the satellite repeats and rDNA of acrocentric chromosomes [18, 19]. This could explain the high frequency of acrocentric chromosomal rearrangements with different partners. Recently some WHS rearrangements have been recognized to involve a translocation between the NOR of an acrocentric chromosome and chromosome 4, producing a satellited 4p chromosome. Some of these cases are sporadic and other familial [2, 7, 10, 20]. Our patients are the product of an adjacent I segregation from the paternal insertion, and the nonaffected girl received both derivative chromosomes by alternate segregation (Figure 4). Wu et al. [10] reported a family with coexisting sibs, which are the products of both types of gametes from an adjacent I segregation: one with a 4p deletion of 5 Mb and classical WHS and the other with a pure duplication of the same region of 4p. 

Only few cases of familiar recurrence of WHS have been described and they are usually associated with a balanced chromosomal translocation in one parent. Nevertheless, the WHS phenotype is modified by the trisomy of other chromosomal region [5, 13, 21, 22]. One instance of a familial recurrence of a 4p pure deletion was due to a meiotic amplification of a maternal 1.5 Mb deletion. The mother had mild WHS, while her two affected sons displayed a typical phenotype. One of the sons was studied and revealed a 2.8 Mb deletion [23]. Another case of two sibs showing a mild form of WHS were reported to have a pure 4p deletion of 2.8 Mb, from a mother with a karyotype 46,XX,t(4;14)(p16.3;p12) [20]. Our patients also have a pure 4p terminal deletion of 6.5 Mb associated with classical WHS phenotype due to a father ins(15;4)(p12;p16.1p16.3); however, only minor phenotype differences were observed between sibs in both families. The differences in the severity of the phenotypes in these two familiar cases could be result of the size of the chromosomal deleted region as has been suggested [5]. 

 The small difference in the size of the deleted material, 25 Kb, among our propositus and his affected sister could be attributed to a maternal polymorphism, recombination aneusomy, or microarray data normalization. The most striking clinical differences between sibs were the type of cardiac defect, the presence of downslanting palpebral fissures and ptosis only in the boy, and kidney malrotation present only in the girl. Comparing the clinical data of our patients with the data reported by Zollino et al. [5] in patients with classical WHS phenotype and deletions between 5 and 18 Mb (Table 1), the only major differences were the absence of ocular coloboma and hypospadias. Until now, few WHS patients had been studied by genomic high-resolution methods [1, 2, 4, 10, 14, 20]. As the number of these studies increases, a better determination of the exact size of deletions will be achieved, improving the definition of the regions and genes implicated in each phenotypic trait associated with the classical WHS.

## 5. Conclusions

The clinical variability in our classical WHS patients could be explained by polymorphisms in the 4p alleles present plus multifactorial inheritance patterns. 

Our results reinforce the importance of thorough clinical diagnosis, as well as conventional and molecular karyotyping of patients and their parents for proper genetic diagnosis and counseling. Particularly, the use of high-density SNP arrays for CNV analysis in the patients enables the determination of the size of the deletion with higher precision and can detect cryptic partial trisomies. In order to give an adequate genetic counseling, the parents of a child with a 4p deletion should always be studied by FISH with subtelomeric 4p and WHSCR1 specific probes, to corroborate if they are carriers of a cryptic balanced rearrangement. In conclusion, we identified a novel type of chromosome rearrangement involved in sibs recurrent classical WHS, and its mechanism is apparently more frequent than previously thought. This case demonstrates the importance of the combined application of classical and molecular techniques to clarify chromosomal structural rearrangements. 

## Figures and Tables

**Table 1:** Phenotype traits of our patients with a 4p deletion of ~6.5 Mb compared with the frequencies of the main clinical features associated with 4p deletions of an average size between 5 and 18 Mb, from Zollino et al. [5].

	II-2	II-4	%
Sex	Male	Female	
Age at examination (years. Months)	9.9	7.5	
Preterm delivery (<38 weeks)	+	+	
Hypotonia	+	+	91
Mild/moderate mental retardation	−	−	24
Severe mental retardation	+	+	80
Seizures	+	+	80
Prenatal growth delay	+	+	84
Postnatal growth delay	+	+	91
Microcephaly	+	+	95
Typical facial dysmorphisms	+	+	100
Cranial asymmetry	+	+	
Round-broad face	+	+	
High-diffuse frontal hair line	+	+	
High forehead	+	+	
Prominent glabella	+	+	
Sparse eyebrows	+	+	
Long eyelashes	+	+	
Downslanting palpebral fissures	+	−	
Ptosis	+^L^	−	
Exophthalmos	+^R^	+	
Ocular coloboma	−	−	30
Strabismus	+	+	
Hypertelorism	+	+	
Broad nasal bridge	+	+	
Beaked nose	+	+	
Short nasal wings	+	+	
Short philtrum	+	+	
Prominent philtrum columns	+	+	
Downturned corners of mouth	+	+	
Cleft lip/palate	+^a^	+^a^	25
Oligodontia	+	+	
Micrognathia	+	+	
Prominent ears	+	+	
Low set and malformed ears	+	+	
Others			
Brain anomalies	+^b^	+^b^	
Hearing loss	+	+	
Congenital heart defects	+^c^	+^d^	52
Renal abnormalities	−	+^e^	37
Hypospadias	−	NA	41
Skeletal anomalies	+^f^	+^f^	37
Sacral dimple	+	+	

Clinical findings: +: present; −: absent; R: right; L: left; NA: not applicable.

^a^Cleft palate.

^b^Cortical/subcortical atrophy, enlargement of lateral ventricles, and septum pellucidum agenesis.

^c^Ventricular septal defect and pulmonary stenosis.

^d^Atrial septal defect.

^e^Malrotation of left kidney.

^f^Hip dislocation.
